# Minimal clinically important difference on the Beck Depression Inventory - II according to the patient's perspective

**DOI:** 10.1017/S0033291715001270

**Published:** 2015-07-13

**Authors:** K. S. Button, D. Kounali, L. Thomas, N. J. Wiles, T. J. Peters, N. J. Welton, A. E. Ades, G. Lewis

**Affiliations:** 1 School of Social and Community Medicine, University of Bristol, Bristol, UK; 2 School of Clinical Sciences, University of Bristol, Bristol, UK; 3 Division of Psychiatry, University College London, London, UK

**Keywords:** Beck Depression Inventory, 2nd edition (BDI-II), depression, minimal clinically important difference, outcome assessment, primary care

## Abstract

**Background:**

The Beck Depression Inventory, 2nd edition (BDI-II) is widely used in research on depression. However, the minimal clinically important difference (MCID) is unknown. MCID can be estimated in several ways. Here we take a patient-centred approach, anchoring the change on the BDI-II to the patient's global report of improvement.

**Method:**

We used data collected (*n* = 1039) from three randomized controlled trials for the management of depression. Improvement on a ‘global rating of change’ question was compared with changes in BDI-II scores using general linear modelling to explore baseline dependency, assessing whether MCID is best measured in absolute terms (i.e. difference) or as percent reduction in scores from baseline (i.e. ratio), and receiver operator characteristics (ROC) to estimate MCID according to the optimal threshold above which individuals report feeling ‘better’.

**Results:**

Improvement in BDI-II scores associated with reporting feeling ‘better’ depended on initial depression severity, and statistical modelling indicated that MCID is best measured on a ratio scale as a percentage reduction of score. We estimated a MCID of a 17.5% reduction in scores from baseline from ROC analyses. The corresponding estimate for individuals with longer duration depression who had not responded to antidepressants was higher at 32%.

**Conclusions:**

MCID on the BDI-II is dependent on baseline severity, is best measured on a ratio scale, and the MCID for treatment-resistant depression is larger than that for more typical depression. This has important implications for clinical trials and practice.

## Introduction

To make informed recommendations about when treatments are of benefit to patients, we must decide what constitutes a clinically important treatment effect. The first step is to know the minimal clinically important difference (MCID) on the outcome measure, i.e. the smallest difference in score considered clinically worthwhile by the patient. MCID is a patient-centred metric that captures both the magnitude of improvement and the value the patient places on that improvement (McGlothlin & Lewis, 2014). Knowing the MCID is crucial for assessing how many additional patients in the treatment arm have achieved this difference, and thus informing whether a treatment is effective in a way that is clinically meaningful to patients. MCID can be determined by clinical consensus, distribution, and anchor-based methods. However, only the last of these methods is truly patient-centred as it anchors change in outcome to the patient's subjective sense of improvement. The Beck Depression Inventory, 2nd edition (BDI-II; Beck et al. 1996) is widely used to assess changes in depressive symptoms in both clinical practice and research. There is, however, limited evidence about the size of the MCID on the BDI-II.

Based on ‘rules of thumb’, the National Institute for Health and Care Excellence (NICE) suggest a difference of ⩾3 BDI-II points is a clinically significant treatment effect for normal depression, and suggest a smaller difference of 2 BDI-II points for treatment-resistant depression (National Institute for Health and Clinical Excellence (NICE) mental health guidelines developed by the National Collaborating Centre for Mental Health (NCCMH), 2004). These influential recommendations have no empirical support. Attempts to empirically determine the MCIDs of other depression and quality-of-life measures have relied upon calculating the difference in symptom scores between groups of people classified as ‘ill’ or ‘well’ (Jacobson & Truax, 1991; McMillan *et al.* 2010). However, this distribution-based method ignores the patients’ own views of improvement, relying instead on the statistical properties of the ‘ill’ or ‘well’ distributions. Such approaches also ignore the potential for baseline dependency; that is that the MCID may vary depending upon how severely ill people are to begin with. For example, an improvement of 3 BDI-II points may be an important improvement for someone with a baseline score of 14, but not for someone with a baseline score of 60. The anchor-based approach compares the outcome measure of interest to an independent and interpretable anchor, such as a global rating of patient improvement. This approach has been used to compare the Hamilton Rating Scale for Depression, and the Japanese version of the BDI-II, to the Clinical Global Impression (CGI) scale (anchor) (Hiroe *et al.* 2005; Furukawa *et al.* 2007). However, the CGI scale is clinician-rated. A more patient-centred approach is to ask the patient to rate his/her improvement on a ‘global rating of change’ scale (Jaeschke *et al.* 1989), and calculate the corresponding difference in score. Walters & Brazier (2003) used this in relation to quality-of-life scales, but we are not aware of a patient-centred anchor-based approach in depression.

### Aim

This study aimed to estimate the MCID on the BDI-II according to a patient-rated global rating of improvement, and assess whether MCID varies by initial severity of depression.

## Method

### Samples

We used data (*n* = 1039) from three large multi-centre randomized controlled trials (RCTs), GenPoD, TREAD, and CoBalT (Table 1) (Lewis *et al.* 2011; Chalder *et al.* 2012; Wiles *et al.* 2013). Each RCT investigated treatment options for depression, used the BDI-II as their main outcome measure, included a ‘Global rating of change’ measure and followed participants over several months providing at least two time periods for analysis. The global ratings of change assessed change relative to the last assessment with a trial researcher, either baseline assessment or previous follow-up visit. Recruited participants in GenPoD and TREAD scored >14 or ⩾14 on the BDI-II, respectively, and fulfilled ICD-10 criteria for a primary diagnosis of depression. The CoBalT study recruited participants with treatment-resistant depression, so in addition to scoring ⩾14 on the BDI-II and fulfilling ICD-10 diagnosis of depression, participants had also adhered to an adequate dose of antidepressant medication for at least 6 weeks prior to entering the trial. The inclusion of CoBalT allowed us to assess the potential differences in MCID for depression that has not responded to antidepressants compared to patients with a new episode of depression (as recruited by GenPoD, TREAD) in primary care.

**Table 1.** Sample characteristics

Study	RCT description	Time period	Time description	*n*	Age, years, mean (s.d.)	Female, *n* (%)	Depression duration >2 years, *n* (%)	BDI-II at first time point, mean (s.d.)	Mean BDI-II change, mean (s.d.)	Better, *n* (%)
GenPod	Reboxetine *v.* citalopram for depression	1	0–6 weeks	332	39.2 (12.7)	216 (65.1)	49 (14.8)	33.4 (9.1)	−14.8 (11.4)	258 (77.7)
		2	6–12 weeks	328	39.6 (12.8)	222 (67.7)	52 (15.9)	19.1 (10.6)	−4.2 (9.1)	240 (73.2)
TREAD	Exercise intervention *v.* care as usual for depression	1	0–4 months	288	40.9 (12.5)	191 (66.3)	33 (11.5)	31.8 (9.3)	−15.3 (11.8)	216 (75.0)
		2	4–8 months	213	42.5 (12.6)	150 (70.4)	24 (11.3)	16.2 (11.7)	−1.2 (9.8)	137 (64.3)
		3	8–12 months	203	42.9 (12.5)	142 (70.0)	25 (12.3)	14.3 (11.4)	−2.0 (8.3)	135 (66.5)
CoBalT	CBT as an adjunct to antidepressants *v*. care as usual for treatment resistant depression	1	0–6 months	418	49.8 (11.8)	307 (73.5)	247 (59.0)	31.6 (10.5)	−9.8 (12.8)	204 (48.9)
		2	6–12 months	385	50.1 (11.7)	285 (74.0)	224 (58.2)	21.4 (13.9)	−2.2 (10.6)	186 (48.3)

RCT, Randomized controlled trial; BDI-II, Beck Depression Inventory, 2nd edition.

Mean BDI-II change corresponds to improvements in depressive symptoms over time in all cases.

Participants in GenPoD were followed for a 12-week period, with follow-up data collection at 6 and 12 weeks. Participants completed the BDI-II and a global rating of change at both follow-ups. Participants in TREAD were followed for a 12-month period, with follow-up data collection points at 4, 8 and 12 months. The BDI-II and a global rating of change measure were collected at all three follow-ups. The CoBalT study followed participants for a 12-month period with data collection points at 3, 6, 9 and 12 months. Global ratings of change were asked at each of these follow-ups, but BDI-II data was collected at the 6- and 12-month follow-ups only. In CoBalT, patients completed the BDI-II and global rating scale as part of the follow-up questionnaire at 6 and 12 months completed primarily during a face-to-face appointment with a researcher. However, participants also completed the global rating scale at 3 and 9 months as part of a follow-up questionnaire administered over the telephone. When answering the global rating of change question at 6 and 12 months, it is likely, therefore, that the majority of patients in CoBalT rated their global change with reference to the telephone follow-up 3 months before. We present participant and study characteristics in Table 1.

### Beck Depression Inventory-II

The BDI-II is a 21-item self-report instrument assessing the common cognitive symptoms of depression, and is considered a valid and reliable instrument for depression screening in the general population (Beck *et al.* 1996; Vanheule *et al.* 2008). The BDI-II is widely used in depression trials. The BDI-II measures the severity of depressive symptoms occurring over the previous 2 weeks, according to DSM-IV criteria (APA, 1994). The items are rated on a 4-point severity scale (0–3) and are summed to give a total score (range 0–63). A higher score on the BDI-II denotes more severe depression.

### Global rating of change

Each of the RCTs included a ‘global rating of change’ question, asking the participants how they felt compared to when they were last seen. In TREAD and CoBalT this question was worded ‘Compared to when we last saw you […], would you say that you are currently feeling better, worse or about the same?’, with participants choosing from three fixed response options: I feel better; I feel about the same; I feel worse. In GenPoD the question ‘How do you feel now, compared to when we last saw you?’ was open-ended, with responses transcribed verbatim. Two researchers (K.S.B. and L.T.) independently rated the open-ended responses. The researchers were blind to the participants’ responses on the BDI-II and other measures. Initially all comments were rated for whether they contained suitable content (0–6 weeks: *n* = 421, kappa = 0.95; 6–12 weeks: kappa = 0.93) and subsequently were classified as follows: feeling much better, better, same, worse or much worse (0–6 weeks: *n* = 332, kappa = 0.89; 6–12 weeks: kappa = 0.92). In total 332 and 328 patients completed the question in time periods 1 and 2, respectively (Table 1).

### Statistical analysis

Analyses were performed using Stata v. 12 (StataCorp., USA) and WinBUGS 1.4.3 (Lunn *et al.* 2000). To explore whether MCID varies according to baseline severity, we assessed change in BDI-II scores as both absolute (difference scale) and relative (ratio scale). As a first step, we stratified by the ‘global rating of change’ to identify the mean difference and proportionate change in symptom scores associated with feeling better, same, and worse (respectively).

#### Generalized linear models (GLMs)

To examine whether MCID varied according to baseline severity of depression, and thus determine whether MCID is best assessed in terms of absolute change or percent reduction in scores from baseline, we used GLMs. Using GLMs we assessed differences in BDI-II change (absolute and ratio) between those reporting feeling better, the same, or worse (McCullagh & Nelder, 1989; Hardin & Hilbe, 2007). The basic GLM used to model observed changes in BDI-II allowed both testing for different link functions (e.g. choice of scale, either absolute or ratio, to measure change) as well as testing for interactions of group differences with baseline. The basic GLM used BDI-II at follow-up as the outcome variable while baseline BDI-II was included as an exposure variable with regression coefficient constrained to one (offset) along with patient's rating groupings and interaction terms of patient's rating grouping with baseline BDI-II. As each study had different treatments and follow-up durations, we treated study as a fixed effect, and thus study specific regression coefficients were included in all models. We also tested for study heterogeneity by calculating χ^2^ (D'Agostino & Weintraub, 1995) and *I*^2^ (Higgins *et al.* 2003) statistics. To assess the most appropriate scale for measuring change (i.e. absolute difference or ratio) we compared the fit of the various models testing for different scales, interaction with baseline and exchangeability assumptions of study-specific effects. We used Akaike's Information Criterion (AIC) as our measure of model fit, with lower values indicating better model fit (Claeskens & Hjort, 2008). In addition to exploring whether MCID was dependent on initial baseline severity, we also examined the effects of individual patient's characteristics such as age, gender, anxiety severity [Clinical Interview Schedule – Revised (CIS-R) score anxiety subscale], and duration of previous depression episodes.

#### Receiver operator characteristic (ROC) analysis

To inform clinical study design and treatment efficacy, we were particularly interested in determining the *minimum* clinically important difference or threshold of improvement above which patients reliably report feeling ‘better’. To this end, we used ROC curves to find the change in BDI-II score (referred to hereafter as the cut-point) that optimally classifies those individuals who felt better and those who did not. Participants were dichotomized into ‘better’ and ‘not better’ using the global rating of change. We generated ROC curves plotting the sensitivity (y-axis) against 1 – specificity (x-axis) for each cut-point on the BDI-II difference (additive scale), and each cut-point on the percent change in BDI-II score from baseline (ratio scale), using the global rating of change as the gold standard. We used the Youden index (Perkins & Schisterman, 2006; Kelly *et al.* 2008), the point on the ROC curve furthest from the line of no discrimination, to determine the optimal cut-point (i.e. the cut-point that maximizes the sum of sensitivity and specificity):

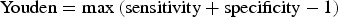
We considered misclassifying more than 30% of patients as better when they were not as clinically unacceptable, and thus added the additional criterion that specificity be ⩾70%, but we also report the analyses without this criterion. We assessed cut-point validity using kappa coefficients to assess the agreement between the dichotomized global ratings and the classifications predicted from the optimal ROC cut-points. This allowed us to compare agreement between measuring BDI-II change in absolute terms using an additive scale, and change relative to baseline using a ratio scale.

## Results

### Sample characteristics

Sample characteristics, according to study, are shown in Table 1. Symptom severity, in terms of mean BDI-II score, was similar at baseline (time point 1) across all three studies, but the mean duration of the current depressive episode was greater in CoBalT. Fewer than 15% of patients in TREAD and GenPoD had been depressed for more than 2 years when they entered the study compared to 59% of patients in CoBalT. Symptoms scores improved (i.e. reduced) most over the first time period. Patients in GenPoD and TREAD showed similar improvements of around 15 BDI-II points. The mean improvement in CoBalT was 10 BDI-II points. A lower proportion of patients in CoBalT reported feeling better relative to those in TREAD and GenPoD at all follow-up points.

### Mean differences and percent reductions stratified by global rating

Table 2 shows the mean change in symptom score, in terms of both differences and percent reduction, stratified by global rating of change, time period and study; the mean improvement in those reporting feeling ‘the same’ was 6.4 BDI-II points (16% reduction), in GenPoD and TREAD and 4.4 BDI-II points (12% reduction) in CoBalT. Regression to the mean is the statistical phenomenon whereby extreme responses measured at time 1 will tend to be less extreme (that is, regress to the mean) at time 2, when participants have been selected on the basis of high scores at time 1. As eligibility criteria favour recruiting individuals who feel at their extreme worst (on a ‘bad day’ a participant may just reach the eligibility threshold, on a ‘good day’ they may not), estimates over the initial time period are most susceptible to this form of bias. There was no evidence of regression to the mean at later time periods. Feeling ‘the same’ was not associated with marked reductions in BDI-II scores at later periods; feeling the ‘same’ in TREAD at time 2 was associated with a slight worsening of symptom scores, otherwise there was marginal change in mean scores in those reporting feeling ‘the same’ at times 2 and 3 (see Table 2). This result was interpreted as reflecting regression to the mean over the first period so all subsequent analysis is restricted to the second time period.

**Table 2.** Descriptive change in BDI-II scores stratified by global rating of change, time period, and study

Time period	Study	*n*	Global rating of change	BDI-II mean change (s.d.)^a^	BDI-II median % change (IQR)^a^
**1**	GENPOD	110	Much better	−20.9 (10.6)	−0.68 (−0.86 to −0.49)
		148	Better	−14.6 (10.2)	−0.40 (−0.86 to −0.49)
		51	Same	−6.4 (8.0)	−0.16 (−0.33 to 0.00)
		19	Worse	−6.9 (9.3)	−0.21 (−0.35 to 0.08)
		4	Much worse	4.5 (7.2)	0.12 (−0.02 to 0.37)
	TREAD	216	Better	−18.9 (10.3)	−0.60 (−0.81 to −0.43)
		60	Same	−6.4 (8.2)	−0.16 (−0.32 to −0.06)
		12	Worse	4.3 (8.1)	0.07 (−0.05 to 0.29)
	CoBalT	205	Better	−17.5 (11.3)	−0.61 (−0.79 to −0.35)
		141	Same	−4.4 (8.7)	−0.12 (−0.29 to 0.05)
		72	Worse	1.2 (9.8)	0.03 (−0.15 to 0.27)
**2**	GENPOD	93	Much better	−9.2 (8.2)	−0.54 (−0.77 to −0.27)
		147	Better	−5.0 (7.6)	−0.27 (−0.53 to 0.00)
		49	Same	−0.08 (6.7)	−0.04 (−0.22 to 0.12)
		37	Worse	4.7 (7.7)	0.16 (0.00 to 0.53)
		2	Much worse	19.5 (29.0)	0.99 (−0.12 to 2.10)
	TREAD	137	Better	−4.3 (8.4)	−0.31 (−0.74 to 0.00)
		54	Same	3.2 (9.5)	0.12 (−0.11 to 0.72)
		22	Worse	7.1 (9.5)	0.23 (0.00 to 0.67)
	CoBalT	186	Better	−6.9 (10.5)	−0.42 (−0.66 to 0.00)
		134	Same	−0.4 (8.0)	0.00 (−0.20 to 20)
		65	Worse	7.3 (7.8)	0.29 (0.07 to 0.75)
3	TREAD	135	Better	−3.9 (7.5)	−0.22 (−0.58 to 0.08)
		49	Same	−0.1 (8.6)	−0.11 (−0.29 to 0.25)
		19	Worse	6.3 (6.8)	0.31 (0.15 to 0.79)

BDI-II, Beck Depression Inventory, 2nd edition; IQR, interquartile range.

a Negative values signify improvement in depressive symptoms; positive values signify worsening in depressive symptoms.

For time periods 2 and 3 those who felt ‘better’ improved on average by 5.0 [95% confidence interval (CI) 3.7–6.2] BDI-II points in GenPoD, 4.3 (95% CI 2.8–5.7) in TREAD and 6.9 (95% CI 5.4–8.4) in CoBalT. A small proportion of patients reported deterioration. For example at time 2, 39 (11%) and 22 (10%) patients reported feeling worse or much worse in GenPod and TREAD, respectively, with a greater proportion feeling worse in CoBalT (*n* = 65, 17%). For time period 2 those who felt ‘worse’ deteriorated on average by 4.7 (95% CI 2.2–7.3) BDI-II points in GenPoD, 7.1 (95% CI 2.9–11.3) in TREAD and 7.3 (95% CI 5.3–9.2) in CoBalT. Reporting improvement and worsening looks relatively symmetrical (Table 2) at time 2; however, low numbers of patients who report feeling worse make formal analyses difficult.

### GLM

**Fig. 1.**
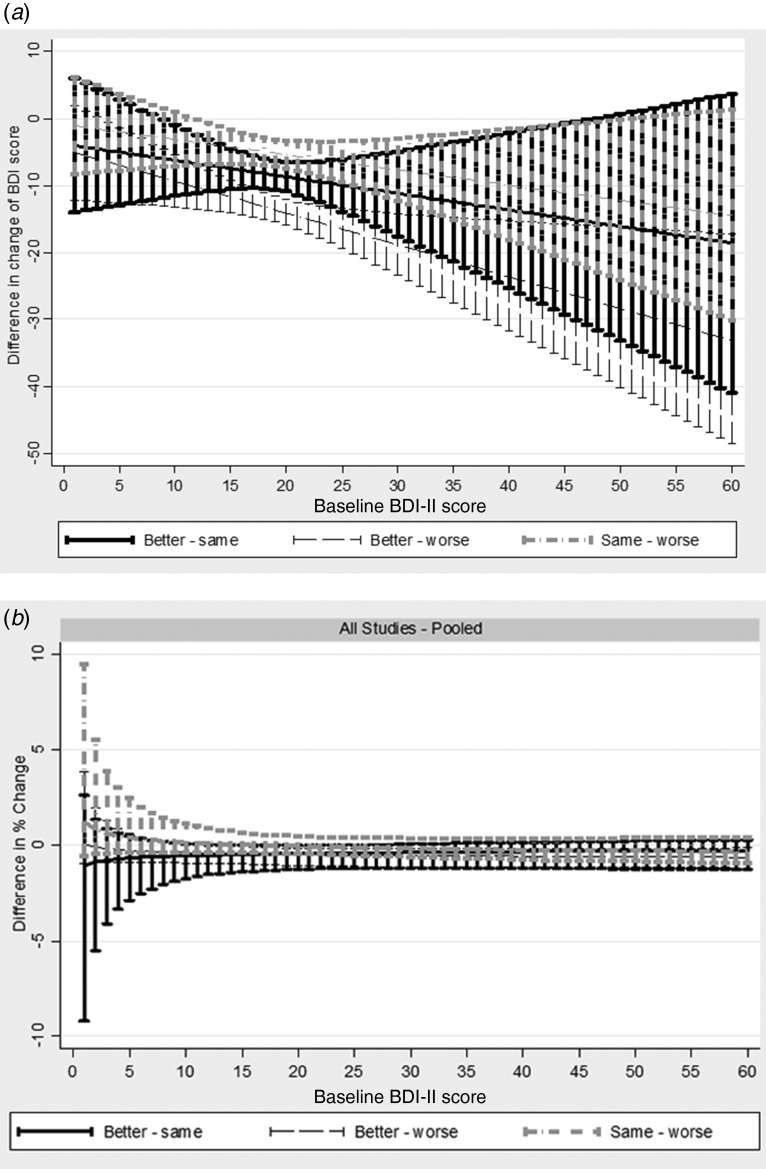
Change in Beck Depression Inventory, 2nd edition (BDI-II) scores by baseline BDI-II score on (*a*) difference scale or (*b*) ratio scale, stratified by global rating from generalized linear model analyses pooled across all studies. In terms of absolute difference (*a*) improvement associated with feeling better increases with increasing baseline severity indicating a baseline dependency. There is less baseline dependency when measuring change in terms of percent reduction from baseline [i.e. ratio scale (*b*)]. In absolute terms, for every ten-point increase in baseline severity, the mean improvement associated with feeling better increased by 4.8 points [interaction term −0.48, 95% confidence interval (CI) −0.85 to −0.09]. The model fit on the absolute scale improved following addition of the interaction with baseline [Akaike's Information Criterion (AIC) reduced from 6649.48 to 6595.81]. Modelling change on the ratio scale (i.e. percent reduction from baseline) further improved the model fit (AIC 6469.01). Adding the interaction into the ratio model improved the model fit (AIC 6371.06), but the interaction with baseline was not supported by the test for interaction (interaction coefficient −0.21, 95% CI −0.65 to 0.23), and the simpler ratio without interaction is more parsimonious.

We found clear evidence of baseline dependency. This is illustrated in Fig. 1*a*. Feeling better was associated with larger improvements (i.e. bigger reductions) in BDI-II score, in absolute terms, as initial severity increased (interaction term −0.48, 95% CI −0.85 to −0.09). For every 10-point increase in baseline severity, the mean improvement associated with feeling better increased by 4.8 points. Furthermore, the model fit on the absolute scale improved following addition of the interaction with baseline (AIC reduced from 6649.48 to 6595.81). Modelling change on the ratio scale (i.e. percent reduction from baseline) further improved the model fit (AIC 6469.01). Adding the interaction into the ratio model improved the model fit (AIC 6371.06), but the interaction with baseline was *not* supported by the test for interaction (interaction coefficient −0.21, 95% CI −0.65 to 0.23), and the simpler ratio without interaction is more parsimonious. This is shown figuratively in Fig. 1*b*. The mean change in those that feel better, same, and worse for the average baseline BDI-II score are provided in Table 3. The mean percentage reduction associated with reporting feeling better was about 36% in GenPod and TREAD and 45% in CoBalT. The mean percentage increase in BDI scores associated with feeling worse was about 14% for GenPod and CoBalT and 18% for TREAD.

**Table 3.** The mean % change from baseline BDI-II according to global rating of change in each study as estimated by the model on the ratio scale without interactions

	% change from baseline	Mean	(s.d.)	2.50%	97.50%
Feeling better	GENPOD	−36.61	(2.28)	−41.07	−32.16
	TREAD	−36.24	(4.06)	−44.28	−28.29
	COBALT	−44.77	(2.68)	−50.02	−39.52
Feeling same	GENPOD	−3.87	(4.53)	−12.63	5.06
	TREAD	1.85	(4.75)	−7.42	11.20
	COBALT	−6.79	(2.46)	−11.60	−1.94
Feeling worse	GENPOD	13.49	(4.85)	3.97	23.05
	TREAD	17.74	(8.06)	2.08	33.62
	COBALT	13.92	(3.69)	6.78	21.17

BDI-II, Beck Depression Inventory, 2nd edition.

We also examined the effects of individual patient characteristics such as age, gender, anxiety severity, and duration of previous depression episodes. We found that the BDI-II scores of older individuals and those with longer duration of previous depression episodes improved less. On average there was a 4% (95% CI 0.5–7.3) reduction in the amount of change from baseline BDI-II for every additional 20 years in age, and a 7.32% (95% CI 0.5–15) reduction in the average change from baseline BDI-II for those with depression duration of >5 years compared to those with <5 years. Age and duration are likely to be confounded. This is consistent with the figures in Table 1 which shows that in the CoBalT sample, where average age and duration is highest, the proportion of individuals reporting feeling better was lowest (48% relative to >64% in GenPod and TREAD). We then examined the effects of age and duration on MCID (by testing for an interaction in the ratio model) and found no evidence to suggest the change in BDI-II associated with feeling better varied by age or duration. We found no evidence that the other patient characteristics were associated with general improvement or MCID.

There was clear evidence for study heterogeneity (χ^2^ = 80.08, df = 2) and high variation of standardized mean differences attributable to heterogeneity (*I*^2^ = 97.5%), with CoBalT being different to both GenPod and TREAD, with the latter two studies behaving similarly.

### ROC analyses

We used ROC analyses to establish the minimum change in BDI-II score required for patients to report feeling better. We present these data in Table 4. On the difference scale, the optimal cut-points were ⩾2, ⩾1, and ⩾3 for GenPoD, TREAD and CoBalT, respectively. The agreement between the classification as better/not better based on these cut-points, and the classifications based on the global rating of change, was reasonable (Table 4). On the ratio scale (i.e. percent reduction in scores relative to baseline), the optimal cut-points were ⩾17, ⩾18, and ⩾32% for GenPod, TREAD and CoBalT, respectively. The agreement assessed using ROC analysis was higher for the ratio scale than the difference scale, suggesting the ratio scale was a better description of data.

**Table 4.** BDI-II cut-points and associated test characteristics for optimization criteria for ROC analyses with specificity ⩾70%.

Study	Sample, *n* (better/not)	Absolute change						Relative change					
		Cut-point	Se	Sp	Correctly classified	AUC (95% CI)	Kappa	Cut-point	Se	Sp	Correctly classified	AUC (95% CI)	Kappa
GenPod	240/88	⩾2	0.75	0.74	74.7%	0.79 (0.7–0.8)	0.43	⩾17%	75	74	75%	0.79 (0.74–0.85)	0.44
TREAD	137/76	⩾1	0.68	0.71	69.0%	0.76 (0.7–0.8)	0.37	⩾18%	61	86	70%	0.76 (0.70–0.82)	0.42
CoBalT	186/199	⩾ 3	0.62	0.71	66.5%	0.75 (0.7–0.8)	0.32	⩾32%	65	86	76%	0.78 (0.73–0.83)	0.52

BDI-II, Beck Depression Inventory, 2nd edition; ROC, receiver operator characteristics; Se, sensitivity; Sp, specificity; AUC, area under curve; CI, confidence interval.

Cut-off values denote improvements in BDI-II scores required for optimal criterion for specificity ⩾70%. Youden Index calculated as *J* = max(sensitivity + specificity − 1). Kappa scores denote agreement between self-reported better/not and ROC classified better/not based on cut-off values.

The results for all analyses were the same irrespective of whether the additional criterion that specificity be ⩾70% was applied, except for the TREAD cut-point on the linear scale; the maximum sum of sensitivity and specificity gave a cut-point of ⩾0 (sensitivity 0.77, specificity 0.63).

## Discussion

To be effective in evaluating the benefits of an intervention for patients, it is necessary to understand what, for patients, constitutes a clinically important treatment effect. Defining ‘clinically important’ is difficult, but one aspect of whether an effect is clinically important is whether the patient reports a global sense of improvement. The BDI-II is widely used in clinical research as an outcome measure of depressive symptoms. We found robust evidence that the improvement associated with reporting feeling better is dependent on initial severity, with larger changes in BDI-II score (in absolute terms) required for those starting at a higher score to feel better (Fig. 1). Furthermore, patients whose symptoms had not responded to antidepressants needed to experience larger improvements on the BDI-II (on average) to report feeling better (Tables 2–4). These finding have important implications for clinical research and practice.

### Strengths and limitations

Our study has a number of strengths, such as the use of robust statistical techniques, and the large sample size provided from the three high-quality RCTs. The ROC analysis provided a means of assessing the optimal cut-point in terms of maximizing the sum of sensitivity and specificity, above which individuals are classified as better. As such the ROC analyses provide the best estimate of the MCID, with the cut-point representing the minimum change required to feel better. The GLM allowed us to investigate model fit associated with improvement in BDI-II as measured on the difference and ratio scales.

The limitations of the study mainly arise from the use of data not designed specifically to address these questions. The global rating of change question used in TREAD and CoBalT was a relatively crude measure, which included only three strata: better; the same; worse. Thus the mean change associated with feeling better in these studies is likely to be an inflated estimate of the MCID. Previous use of global ratings of change to assess MCID on a quality-of-life measure (Walters & Brazier, 2003) used a global rating that included: much better, somewhat better, about the same, somewhat worse, and much worse. We know little about the reliability of the global rating question. Work is needed to address this. Asking the patient how they feel has obvious face validity and is a reasonable starting point to inform MCID. Furthermore, there is little agreement between patient and clinician global ratings (Forkmann *et al.* 2011), with patient ratings performing better at tracking recurrence in maintenance therapy (Dunlop *et al.* 2011). The lack of agreement between clinician and patient global ratings has led to recommendations that both should be included in RCTs (Forkmann *et al.* 2011). However, there is a move away from costly clinician assessments towards patient-reported outcomes, especially in large-scale pragmatic trials in primary care. The evidence to support the use of patient-reported outcomes in routine practice remains largely unexplored despite the growing need to integrate and understand them especially in the context of primary care (Fitzpatrick, 1998). Our work offers and initial step in understanding MCID on the patient-reported BDI-II.

Each of the studies used different follow-up periods. Therefore, study is confounded by time to follow-up, as well as other study design specifics, making it difficult to disentangle the effects of time from the more interesting characteristics of the patient population. However, we obtained similar results for GenPod and TREAD despite their differing follow-up periods suggesting that there are no major effects of time between 6 weeks and 4 months follow-up, when study populations are similar.

We measure MCID as a within-person difference, to inform between-group differences that are important for considering treatment effects in study design. Although not necessarily identical, by developing the MCID with reference to individuals’ perceptions of their own improvement, our results can inform such target group differences. Finally, it is unclear how clinically important it is to report feeling better, and feeling better is likely to be one of several aspects of clinical importance. Further research is needed to explore other indexes, such as family or clinician ratings of global improvement. However, patients seek help in the main to ‘feel better’ so our approach accords with their views.

### Baseline dependency

The main finding from our study is that the improvement in BDI-II scores associated with reporting feeling better is dependent on initial severity. Classifying individuals as better or not on the ratio scale produced better agreement with patients’ reports relative to the difference scale in the ROC analyses. This was supported by the GLM analyses where overall model fit was worst for the model on the difference scale without the baseline interaction term, improved following the addition of the baseline interaction term, and improved further still using the ratio scale. Therefore, modelling change on the ratio scale results in better calibrated models, as well as conferring interpretative advantages, removing the need to incorporate interaction terms. We suggest, therefore, that MICD is best assessed on the ratio scale (i.e. percent reduction in scores from baseline).

### MCID

The cut-point from the ROC analyses provides the optimal threshold (in terms of maximizing the sum of sensitivity and specificity) above which individuals are classified as ‘better’ and below which they are classified as ‘not better’. The ROC analyses therefore likely provide the best estimates of MCID, with an improvement from baseline of 17%, 18% and 32% for GenPod, TREAD and CoBalT, respectively. The mean improvement associated with feeling better (estimated for those with average baseline scores form the GLM analyses) was 37%, 36% and 45% for the three trials, respectively. Consistent with the GLM model, there was greater agreement between self-reported ‘better/not’ and ROC-classified ‘better/not’ for proportional cut-off values (i.e. ratio scale) relative to absolute values (Table 4).

We have focused on MCIDs in terms of benefit; however, it is worth considering the minimum change associated with deterioration. Relatively few patients reported feeling worse which increased uncertainty in estimates. The mean change in BDI-II stratified by global rating looks relatively symmetrical on the absolute scale, but the magnitude of change to report feeling worse seems smaller relative to feeling better on the ratio scale (Table 2). This is consistent with the 14–17% percentage increase in BDI-II scores associated with feeling worse, compared to 36–45% mean improvement associated with feeling better, as estimated by the GLM model (Table 3).

### Comparing study populations

The findings from all analyses indicated that the individuals with more chronic depression, such those recruited into CoBalT who had not responded to antidepressant treatment, require a larger reduction in BDI-II score to report feeling better, and the GLM analysis provided statistical evidence that this group was distinct from the GenPoD and TREAD samples. The more chronically ill CoBalT sample seemed less affected by regression to the mean during period 1. This implies that levels of depression are more stable in the CoBalT sample, which is expected given their resistance to antidepressant treatment and the chronicity of their depression. We also explored whether patients characteristics such as age, gender, anxiety severity, and duration of previous depression episodes affected MCID estimates but found little evidence that these influenced change associated with feeling better. Given the inter-cohort variability in MCID, further work is needed to explore MCID in other populations such as co-morbid, geriatric, and adolescent depression.

### Research implications

Our finding, that clinically important improvement is dependent on initial depression severity, has important implications for clinical research. It indicates that the NICE guidance suggesting a change of ⩾3 BDI-II points as clinically important is unhelpful as it does not account for baseline dependency. According to our results, a between-group difference (i.e. treatment effect) of 3 BDI-II points would be trivial in a sample with an average BDI-II score of 60, but more relevant in a sample averaging BDI-II scores of 14.

The need to account for baseline dependency impacts on the assessment of treatment effects, in particular it has implications for power calculations. One approach might be to power studies to detect difference in mean BDI-II scores corresponding to the average MCID based on expected baseline BDI scores expected in the study being designed. For example, the average MCID (assuming an MICD of 18%) in the linear scale in a sample with average BDI-II scores of 33 is 5.94, and the study could be powered to detect this treatment effect. This approach assumes that the treatment effects are additive on a linear scale. However, if treatment effects also act multiplicatively, and therefore are best measured on the ratio scale, then BDI scores measured in RCTs could be analysed after log transformation, and powered on an 18% reduction of scores. The results from this paper suggest a multiplicative (i.e. ratio) relationship for MCID. Whether this extends to treatment effects has yet to be investigated, and research is needed to explore this, which could have major consequences for how RCTs for the management of depression are powered and analysed.

The NICE guidelines suggest the *smaller* criterion for clinically important differences of 2 BDI-II points for treatment-resistant depression compared to a criterion of 3 points in new episodes of depression (NICE, 2004). Although the NICE guidelines refer to treatment effects, the direction of effect is the opposite of the results that we have reported here, in that patients who had not responded to antidepressants require a *larger* proportionate change in BDI-II score to report an improvement. This should be borne in mind when designing clinical trials, and interpreting clinical results in this population. However, the differences in MCID might also reflect an underlying cognitive bias, such as more hopelessness, leading patients with longer depression duration and non-response to antidepressants to be less inclined to report feeling an improvement. Further research is needed to investigate this.

Finally, there was significant between study variability. For the purpose of establishing a widely accepted MCID an evidence synthesis approach may be needed. The inferential objectives of such an exercise require synthesising evidence about the changes or improvements experienced by patients from different studies (wide population base with broad distribution of baseline values – usually these baseline distributions have limited range in published RCTs), multiple waves (to estimate change more precisely), integrating multiple measurements/instruments (to control for measurement error in each occasion) and integrating different perspectives (clinical improvements and patient-centred reports). The hierarchical GLM approach we used here could be used as a template to build upon, explore and integrate the different sources of variability that affect the distribution of change for evaluable groups by different methods (Pepe, 2003).

### Clinical implications

Within the Improving Access to Psychological Therapies (IAPT ) framework, outcome data, such as scores of the Patient Health Question (PHQ-9), are increasingly being used to monitor improvement during treatment. If our results generalize to other measures of depression, they may indicate the need to assess improvement on the ratio scale, i.e. percent reduction in scores from baseline. Whether baseline dependency holds for outcome measures other than the BDI-II remains to be seen, and further work is needed. The possible impact of measurement error also needs investigation. Understanding what, for patients, constitutes a clinically important improvement is vital for effective clinical research and practice. We have estimated the change in BDI-II scores associated with feeling better. Qualitative methods may help us to explore the concept of feeling better, and may inform what other indexes, such as corresponding change on quality of life measures, contribute to an effect being clinically important. Finally, we use the single global rating question as the reference to inform what change on average is required to feel better. In terms of clinical consultation and the individual patient, we would expect a scale with multiple items to be more sensitive to change, than a single item (although work is needed to assess this). Therefore we do not advocate replacing a multi-item scale such as the BDI-II or PHQ-9 with a single global question, although a clinician may wish to get an overall sense of improvement in addition to a more detailed assessment of symptoms.
